# Supplementary material for: Tumor-Targeted Delivery of the p53-Activating Peptide VIP116 with PEG-Stabilized Lipodisks
Source: Nanomaterials (Basel). 2020 Apr 19;10(4):783. doi: 10.3390/nano10040783 (PMC7221704; doi:10.3390/nano10040783)
Supplement: Supplementary file 1 [file nanomaterials-10-00783-s001.pdf]

Supplementary

Tumor-targeted delivery of the p53-activating peptide VIP116 with PEG-stabilized lipodisks

Sara Lundsten [1], Víctor Agmo Hernández [2,3], Lars Gedda [2], Tina Sarén [1], Christopher J. Brown [4], David P. Lane [4,5], Katarina Edwards [2,†] and Marika Nestor [1,*,†]

[1] Department of Immunology, Genetics and Pathology, Uppsala University, 751 85 Uppsala, Sweden; sara.lundsten@igp.uu.se (S.L.); tina.saren@igp.uu.se (T.S.)
[2] Department of Chemistry—BMC, Uppsala University, 751 24 Uppsala, Sweden; victor.agmo@kemi.uu.se (V.A.H.); lars.gedda@kemi.uu.se (L.G.); katarina.edwards@kemi.uu.se (K.E.)
[3] Department of Pharmacy, Uppsala University, 751 23 Uppsala, Sweden
[4] p53 lab, Agency for Science, Technology and Research (A*STAR), Singapore 138648, Singapore; cjbrown@p53lab.a-star.edu.sg (C.J.B.), dplane@p53lab.a-star.edu.sg (D.P.L.)
[5] Department of Microbiology, Tumor and Cell Biology, Science for Life Laboratory, Karolinska Institutet, 171 65 Solna, Stockholm, Sweden
* Correspondence: marika.nestor@igp.uu.se; Tel.: +46-70234-1881
† Shared senior authorship.

Materials and Methods

Cryo-TEM

The presence of lipodisks was verified with cryo-transmission electron microscopy (cryo-TEM) using a Zeiss Libra 120 Transmission Electron Microscope (Carl Zeiss NTS, Oberkochen, Germany) according to previously described protocol [21] Figure S1. The samples were equilibrated at 25 °C and high relative humidity (>90%) within a climate chamber, prior to vitrification. The microscope was operating at 80 kV and in zero loss bright-field mode. Digital images were recorded with a BioVision Pro-SM Slow Scan CCD camera (Proscan GmbH, Scheuring, Germany) and iTEM software (Olympus Soft Imaging System GmbH, Münster, Germany).

QCM-D

A quartz crystal microbalance with dissipation monitoring (QCM-D) was used to study the interaction of VIP116 with lipodisks and lipid membrane. Silica or biotynilated gold sensors (Q-Sense, Gothenburg, Sweden) was mounted on a QCM-D E1 (Q-sense) instrument thermostatted at 21 °C. Frequency and dissipation data were collected at the 3rd, 5th, 7th, 9th, 11th and 13th overtones and the immobilized mass was calculated as previously described [22]. Matlab R2016b (The MathWorks, Natick, MA, USA) was used for performing the calculations. A constant flow rate of 100 µL/min was used throughout the interaction assays.

For immobilized lipodisks, biotinylated gold sensors were first coated with streptavidin and subsequently biotinylated lipodisks. The system was then equilibrated with 0.5% DMSO in PBS pH 7.4 which was used throughout the assay. VIP116 with concentrations of 10, 30 and 100 nM was stepwise added. After the association phase, 0.5% DMSO in PBS was applied to study dissociation. To verify that VIP116 did not unspecifically bind to the bare sensor, a control experiment was performed without lipodisks (Supplementary Materials Figure S2). Kinetics constants were calculated by fitting the real-time R_{eff} data to a Langmuir 1:1 binding model with TraceDrawer (Ridgeview Instruments, Vänge, Sweden).

For the supported lipid bilayer in gel phase, silica sensors were first cleaned according to the instructions of the provider. (10 min UV/ozone treatment followed by immersion in SDS 2% and finally 10 min UV/ozone treatment). The sensor were then mounted on the instrument and exposed to a continuous flow of PEGylated DPPC liposomes in PBS (~1 mg/mL) until the surface was saturated. The

system was rinsed with dearated PBS, before the flow was stopped and a temperature program (a constant increase from 21 °C to 60 °C for 70 min, a temperature plateau of 60 °C for 25 min and a decrease back to 21 °C for 70 min) was applied and looped overnight to cause the liposomes to collapse and form a lipid bilayer in gel phase as confirmed by the final frequency change (−33 Hz) value and a very low dissipation factor ($<4 \times 10^{-6}$). After formation of the bilayer, the binding assay was performed with stepwise addition of 10, 30 and 100 nM VIP116. The R_{eff} at equilibrium for each concentration (average of last 100 data points before addition of next concentration or start of dissociation) for the VIP116-lipid bilayer interaction was compared to that of the VIP116-lipodisk interaction. For the VIP116-lipodisk interaction, the data was fitted to a Langmuir 1:1 model (one site—specific binding) to calculate kinetic parameters.

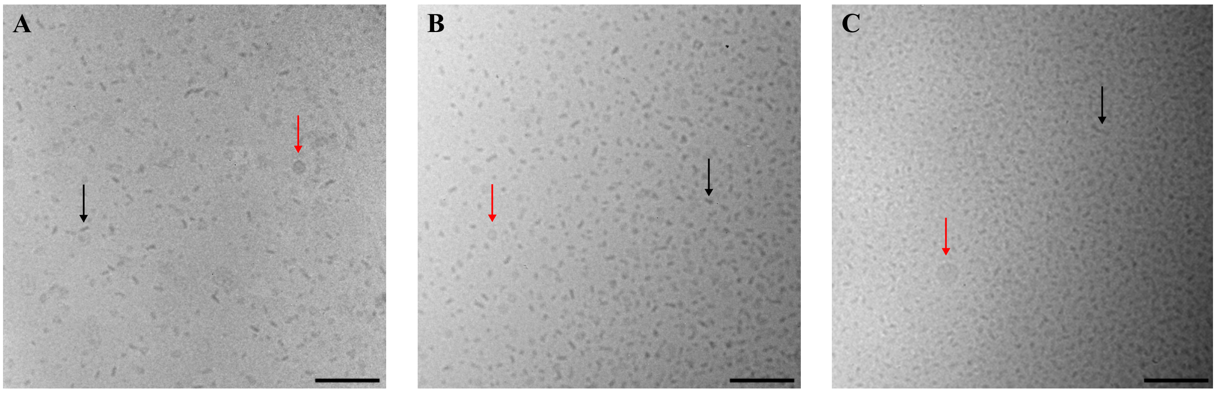

Figure S1. Cryo-TEM of lipodisks used for (**a**) QCM-D as well as (**b**) targeting and (**c**) non-targeting lipodisks for cell assays. Black arrows = edge-on, red arrows = face-on. Bar = 100 nm.

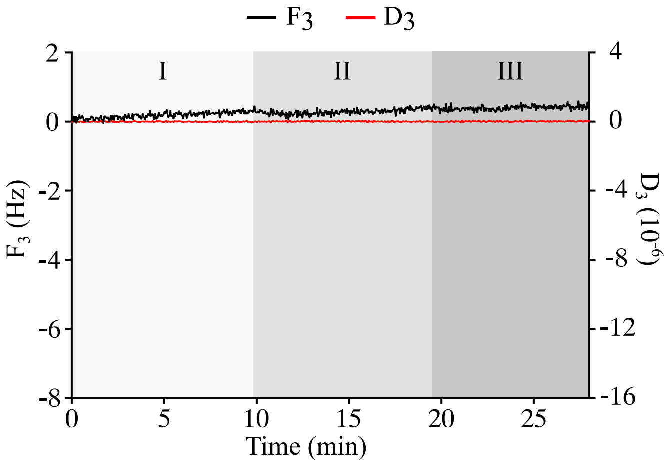

Figure S2. Unspecific binding of VIP116 to a biotinylated gold-sensor coated with streptavidin. Raw data displaying third overtone of frequency (black) and dissipation (red) with base line (I), 30 (II) and 100 (III) nM VIP116.
